# The Folding of *de Novo* Designed Protein DS119 via Molecular Dynamics Simulations

**DOI:** 10.3390/ijms17050612

**Published:** 2016-04-26

**Authors:** Moye Wang, Jie Hu, Zhuqing Zhang

**Affiliations:** College of Life Sciences, University of Chinese Academy of Sciences, Beijing 100049, China; wangmoye13@mails.ucas.ac.cn (M.W.); hujie14@mails.ucas.ac.cn (J.H.)

**Keywords:** protein folding, DS119, molecular dynamics simulation, transient dimer, intermediate states

## Abstract

As they are not subjected to natural selection process, *de novo* designed proteins usually fold in a manner different from natural proteins. Recently, a *de novo* designed mini-protein DS119, with a βαβ motif and 36 amino acids, has folded unusually slowly in experiments, and transient dimers have been detected in the folding process. Here, by means of all-atom replica exchange molecular dynamics (REMD) simulations, several comparably stable intermediate states were observed on the folding free-energy landscape of DS119. Conventional molecular dynamics (CMD) simulations showed that when two unfolded DS119 proteins bound together, most binding sites of dimeric aggregates were located at the N-terminal segment, especially residues 5–10, which were supposed to form β-sheet with its own C-terminal segment. Furthermore, a large percentage of individual proteins in the dimeric aggregates adopted conformations similar to those in the intermediate states observed in REMD simulations. These results indicate that, during the folding process, DS119 can easily become trapped in intermediate states. Then, with diffusion, a transient dimer would be formed and stabilized with the binding interface located at N-terminals. This means that it could not quickly fold to the native structure. The complicated folding manner of DS119 implies the important influence of natural selection on protein-folding kinetics, and more improvement should be achieved in rational protein design.

## 1. Introduction

In a cellular environment, most proteins must fold into defined three-dimensional (3D) structures to gain functional activity [1]. Some proteins accomplish the folding process under the assistance of molecular chaperones [2,3], and others can finish this process by self-assembling. To study protein folding is to understand how proteins fold spontaneously onto their specific, biologically functional 3D structures. Aberrant folding behavior will cause proteins to become degraded or to aggregate into toxic species, which is associated with diseases such as type 2 diabetes, Alzheimer’s disease, and Parkinson’s disease [4]. Therefore, research on protein folding mechanisms is of great significance to explore the pathogenesis of “folding disease”, for the further prevention and cure of these diseases.

Over several decades of this study, one fundamental feature has been found, namely, that many naturally evolved single-domain proteins fold cooperatively in a two-state-like manner [5,6], which is suggested as serving crucial functions, such as avoiding harmful aggregation, and might be the result of biological evolution [7,8]. More recently, through all-atom molecular dynamics simulations, Shaw and coworkers studied more than 10 relatively small proteins and observed many folding and unfolding transitions in long equilibrium trajectories [9,10], which provided much more atomistic details of folding kinetics and mechanisms. The results indicated that this technique could serve as a powerful tool for elucidating protein folding behavior.

Compared to natural proteins, *de novo* designed proteins are usually not subjected to the process of natural selection. From the first computer-generated *de novo* designed mini-protein FSD-1 [11] to recent *de novo* designed repeat proteins and the TIM-barrel protein [12,13], more and more proteins have been successfully *de novo* designed [14,15,16]. Although their folds are close to target structures and usually stable, these novel proteins have exhibited quite different folding characteristics from those of natural proteins. For example, the first computer-generated *de novo* designed mini-protein FSD-1 with aββα fold [11], and its analog FSD-1ss, both display weakly cooperative folding behavior [17,18]. Another *de novo* designed three-helix bundle protein α3D was found to fold at an ultrafast rate [19], and a recent study indicates that, unlike other natural proteins, nonnative contacts play an apparent role during its folding process [20]. Additionally, *de novo* designed protein Top7, with a novel target structure and sequence [14], folds in a significantly less cooperative way [21]; further coarse-grained simulations revealed that its complex folding kinetics might arise from its native topology [22], and nonnative hydrophobic interactions caused it to become trapped in metastable states [23]. All these investigations indicate that, without evolution information involved, *de novo* designed proteins fold in different manners. The investigation of the folding mechanism of these proteins would help in the reconsideration of design strategy in order to make designed proteins more natural.

In this study, we focus on a recently *de novo* designed βαβ motif DS119, which has 36 amino acids (sequence: GSGQVRTIWVGGTPEELKKLKEEAKKANIRVTFWGD) with two parallel β-strands packed against one α-helix [24]. Experiments exhibited that it has highly thermal stability with *T*_m_ larger than 80° [24]. Molecular simulation studies predicted that it would fold in a timescale of µs [25], while recent experiments demonstrated that it folds unexpectedly slowly in a timescale of seconds [26]. Furthermore, fluorescence resonance energy transfer (FRET) and chemical cross-linking measurements detected transient dimers during the slow folding process [26]. Why does such a small protein fold so slowly? What kind of transient dimer is formed and how does it influence the folding process? Here, we investigate possible reasons by means of all-atom molecular dynamics simulations. Firstly, we used replica exchange molecular dynamics (REMD) [27], which has been extensively applied in many systems such as short peptide aggregates, intrinsic disorder proteins, and small single-domain globular proteins, as it has an advanced sampling capability [28,29,30,31] to obtain free-energy landscapes for the single protein DS119. Then, eight conventional molecular dynamics (CMD) simulations were conducted to detect how two unfolded DS119 proteins aggregate. The folding free-energy landscapes exhibited both in folding and unfolding conditions, intermediate states were obviously observed. The early initial aggregation stage of two proteins showed that the N-terminal segment might be critical for forming transient aggregates, and individual DS119 in the aggregates might be trapped in its intermediate states. The present work of the folding mechanism of DS119 would not only complement the understanding of protein folding, but also provide insights into improving rational protein design. Furthermore, the investigation of transient dimer formation could provide potential clues to intervening with protein aggregation, which is associated with misfolding diseases.

## 2. Results

### 2.1. Folding Free Energy Landscapes of DS119

The folding free-energy profiles of DS119 were obtained via REMD in this study. By exploring conformations at different temperatures and overcoming energy barriers through replica exchange, REMD has become a popular technique used to enhance sampling in biomolecule systems. Starting from a fully extended conformation of protein DS119, a 500 ps molecular dynamics (MD) simulation was conducted at 500 K. After energy minimization, the structure was adopted as the initial conformation for the 26 replicas in REMD (see Materials and Methods for details). The simulation of each replica was performed for 500 ns, and we collected the conformations sampled after 50 ns for analysis.

**Figure 1:**
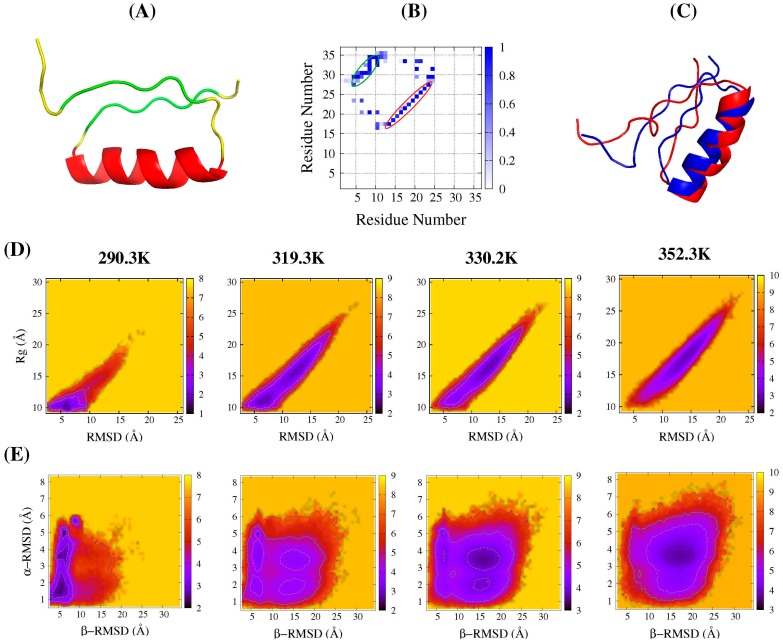
DS119 structures and its folding free-energy landscapes from replica exchange molecular dynamics (REMD) simulations. (**A**) The first NMR frame of DS119 (PDB ID: 2KI0). The α-helix (Pro14-Ala27) is colored with red, and the β-sheet (Val5-Val10 and Ile29-Trp34) is colored with green, the loop and turn regions are colored with yellow; (**B**) The residue contact frequency map based on 20 NMR structures of DS119. The regions encircled by red and green lines correspond to the α-helix and β-sheet shown in (**A**), respectively; (**C**) Alignment of the first NMR structure (in blue) and the best simulated structure (in red) from REMD; (**D**,**E**) Free energy landscapes as the function of different coordinates at typical temperatures (290.3 K, 319.3 K, 330.2 K, and 352.3 K from **left** to **right**). The reaction coordinates are: the root-mean-square deviation (RMSD) and the radius of gyration (*R*g) in (**D**); and the α-helix RMSD and β-sheet RMSD in (**E**).

We firstly monitored root-mean-square deviation (RMSD) and the radius of gyration (*R*g). The most native-like conformation had a RMSD of 3.08 Å (the aligned structures shown in Figure 1C) at temperature 297.8 K. Figure 1D showed the folding free-energy profiles at four typical temperatures. At near-room temperature (290.3 K), the sampled stable state was located at a RMSD of around 6 Å and a *R*g of around 10 Å, and we called it a near-folded state. With the increase of temperature, another minimal region appeared apparently with a larger *R*g and RMSD, which implied the occurrence of unfolding. At 319.3 K, the two areas were both observed to be well-populated; until 352.3 K, most of the populated conformations were located in the unfolded region. Therefore, only the near-folded and unfolded states were observed in the *R*g-RMSD two-dimensional (2D) profiles. The transition occurred at around 319 K, some lower than that reported in experiments [24].

Further, segment RMSDs were monitored to conduct DS119 folding free-energy landscapes, which have been used in many other studies for a deeper explanation of protein folding [30,32]. Here, the α-RMSD and β-RMSD were measured. As shown in Figure 1E, even at near-room temperature (290.3 K), three states were evidently observed, with the similar β-RMSD around 5.4 Å, but quite diverse α-RMSDs, around 1.5, 3.5, and 5.0 Å, respectively. It indicated that the N- and C-terminal parts might have already approached each other, whereas the helix was formed in different native-like degree. With the increase in temperature, the transition to the unfolded state occurred at around 319.3 K—same as that in the *R*g-RMSD profiles. At this temperature, besides the unfolded minimal region and the three ones mentioned above, another region was also identified, with the β-RMSD of 15.0 Å similar to the unfolded state, but a smaller α-RMSD of around 2.0 Å, which implied that, in this intermediate state, α-helix was formed quite well. The further increase in temperature facilitated the gradual shifting of the conformation population toward the unfolded state, as shown at 330.2 and 352.3 K.

Comparing Figure 1D and E, only two states emerged onto the profiles based on *R*g-RMSD, while the profiles based on α-RMSD and β-RMSD revealed several evident intermediate states; this suggests that different order parameters could give different information toward our understanding of the folding landscape. Therefore, we further measured some other order parameters, such as the distance of aromatic groups between Trp9 and Trp34 (W9-W34 distance), as the aromatic interaction was indicated as being quite important for stabilizing the folded structure during the design [24] as well as the solvent-accessible surface area (SASA) of the hydrophobic core. The 2D profiles obtained by the combination of different order parameters are shown in Figure S1 of the supplementary materials. The profiles containing the W9-W34 distance showed a similar character to those containing β-RMSD, as both monitor the N- and C-terminal interactions. Profiles based on SASA of the hydrophobic core displayed attributes similar to those based on *R*g, as both are related to the degree of conformation extension. Another order parameter, the helix content was also monitored to conduct the profiles *versus* the W9-W34 distance, which display a similar folding landscape feature with that shown in Figure 1E.

### 2.2. Structure Features of Intermediate States

**Figure 2:**
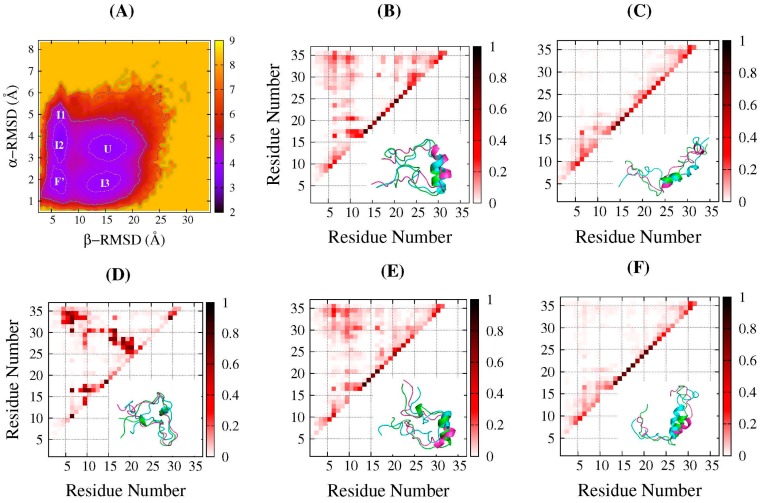
The structural characterization of different states at 319.3 K from REMD simulations. (**A**) The free-energy landscape at 319.3 K as a function of the α-helix RMSD and β-sheet RMSD (same as Figure 1E). Five observed minimal regions were marked as native-like state (F’), unfolded state (U), and three intermediate states (I1, I2 and I3); (**B**) **Top left**: The intra-chain residue contact frequency map for native-like state region (F’) in (**A**). **Bottom right**: The aligned three representative structures; (**C**–**F**) are the same as (**B**) but represent U, I1, I2, and I3 regions in (**A**).

In order to explore the structural features of the intermediate states in more detail, we focused on the folding free-energy landscape based on α-RMSD and β-RMSD at near-transition temperature (319.3 K). We named the five minimal regions as F’ state, U state, and I1, I2, and I3 states respectively marked in Figure 2A. The intra-chain residue contact probability maps, based on at least 100 representative frames sampled in the corresponding regions with a time step interval of at least 100 ps, were presented in the top-left triangular areas in Figure 2B–F. Here, the contact was defined when the distance between the two heavy atoms in each residues was less than 4.5 Å, and the two residues were separated by at least 3 residues along the sequence. Furthermore, three of the sampled frames were selected randomly, and the aligned schematic structures were shown in the bottom-right triangular areas of Figure 2B–F. The F’ state was centered at around (6.0, 1.8 Å), with a smaller β-RMSD and α-RMSD; therefore, it was the most native-like. In Figure 2B, the residues from 14 to 27 showed a higher probability of forming an α-helix, and the aligned schematic structures confirmed that. The points in top-left corner indicated that N- and C-terminals had contacts, and the whole protein collapsed as the *R*g reached approximately 10 Å (Figure 1D). The β-sheet was almost not formed, since most of the contacts were nonnative, as the aligned structures shown in Figure 2B indicate. In short, in this native-like state, the main deviation from the native structure occurred on β-sheet segments. The unfolded region U exhibited an extended structural feature, with the β-RMSD at around 15 Å and very low N-C terminal contact probability, as shown in Figure 2C. In this region, some short α-helices could still be identified with an α-RMSD value close to 3~4 Å, but most occurred to the residues 14 to 17, although the location apparently varied. This indicates that, in the unfolded state, the conformations are not completely unstructured.

Regions I1-I3 in Figure 2A were three intermediate states. Among them, I1 and I2 emerged in folding conditions at relatively lower temperatures along with the native-like F’ state, while I3 was observed when the temperature increased and accompanied the unfolding state U. The contact probability maps of I1, I2, and I3 were shown in Figure 2D–F. Comparing Figure 2D,E, the major difference between I1 and I2 is the extent of the α-helix formed; as in the former, only very scattered short helices were detected, but in the latter, more residues were involved in the helix formation, especially residues 14–27, similar to the U state. Similar to the F’ state, both regions showed a β-RMSD at around 6.0 Å, exhibiting that N- and C-terminals were close to each other but formed nonnative contacts. Region I3 was centered around at (15.0, 1.9 Å), with a similar α-RMSD to that of the native-like state F’, and a similar β-RMSD to that of the unfolded state U. According to the probability contact map and the aligned schematic structures in Figure 2F exhibited in this ensemble, the residue 14 to 27 formed α-helix quite well, while the two terminals completely separated.

The free-energy minimal values of the populated regions, as well as the barriers between them in Figure 2A, were estimated to assess their thermodynamics stability. For the regions I1, I2, and F’, the free-energy minimum were 2.96, 3.33 and 3.72 kal/mol, respectively. The barrier from I1 to I2 was 0.99 kal/mol, and, from I2 to F’, was 0.68 kcal/mol. For the regions I3 and U in unfolding condition at higher temperatures, the free-energy minimums were 3.62 and 3.81 kal/mol, respectively, with a barrier of 0.5 kal/mol from the former to the latter. Therefore, these intermediate states all have comparable stabilities with the native-like state or the unfolded state, and the free-energy barriers between them are quite low, ranging from 0.5 to 1.0 kal/mol. This means that, during the folding of DS119, it might be quite easy to become trapped in the intermediate states due to the roughness of the free-energy landscape, which might result in an inability to quickly fold as natural proteins can.

### 2.3. Common Contact Pattern of Initial Dimeric Aggregates

**Figure 3:**
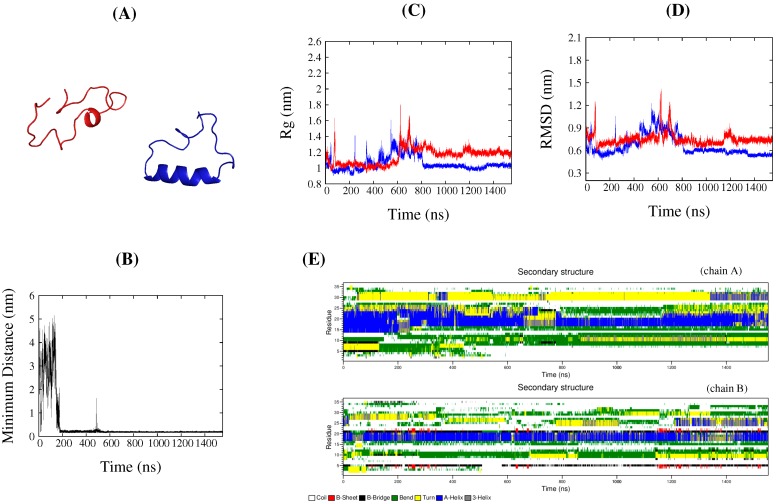
Analysis for a representative trajectory (simulation 1) from conventional molecular dynamics (CMD) simulations. (**A**) Typical initial structure for a dimer aggregation simulation; (**B**) Minimal distance of the two proteins *versus* simulation time; (**C**) *R*g values of each protein along with simulation time; (**D**) RMSD values of each protein along with simulation time; (**E**) Secondary structure analysis (based on a dictionary of secondary structure of proteins (DSSP)) of two proteins; in (**C**,**D**), the blue lines denote chain A, and the red ones denote chain B.

We used all-atom CMD simulations to detect the features that initial dimeric aggregates might possess, because experiments have demonstrated that transient dimers form during the folding process of DS119 [24]. In the experiments, the entire folding process, including two protein associations followed by disassociation, took several seconds to occur. It has been impossible for CMD simulations to match such a time scale to date. In this study, we only considered the very initial stage of the aggregation to detect how the aggregates form in the beginning. Eight independent CMD simulations were performed, and each of them started from two unstructured DS119 monomers, separated by at least 10 Å, as shown in Figure 3A. The simulations were conducted by using the Gromacs4.5.6 package [33]; more details of the simulation protocols can be found in the Materials and Methods section. The minimum distance between the two monomers in Figure 3B and Figure S2 show that dimeric aggregates were observed in seven of the eight simulations, with binding time starting from 30 to 750 ns. Therefore, in the following section, our analysis will focus on the seven simulations in which dimer aggregation occurred.

The parameters *R*g, RMSD, and the secondary structure assessment for each protein were monitored, as shown in Figure 3C–E for the typical trajectory of simulation 1, and Figures S3–S5 for other simulations. The figures demonstrate that, after the aggregation occurred, the *R*g varied from 9 to 18 Å, and, in some simulations (Figure S3A,D,F), one monomer had a more extended conformation than the other in the aggregates. The RMSDs also varied largely, from 3 to 20 Å, with some monomers displaying a larger value than the others in some simulations (Figure S4A,E,F). The secondary structure was assessed based on a dictionary of secondary structure of proteins (DSSP) algorithm [34]. Figure 3E and Figure S5 exhibited the DSSP analysis result, with a short or long helix observed for quite a long time, but scattered nonnative β structures emerged from only a few simulations. Apparently, no folded structures were observed in any of the aggregation simulations.

**Figure 4:**
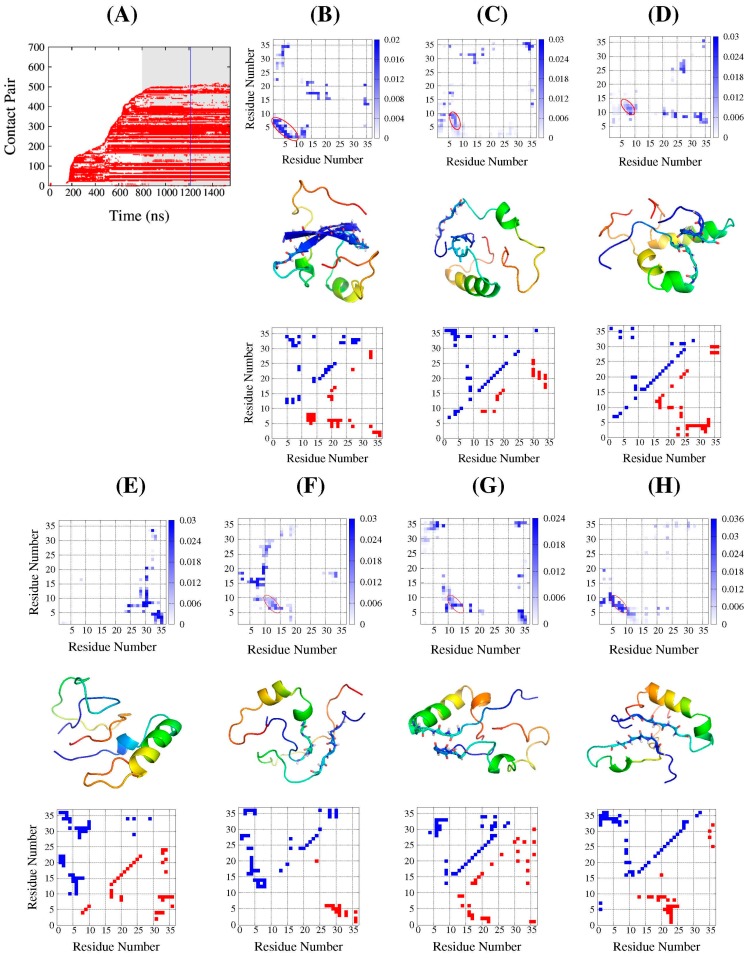
Contact patterns and structural characterization in the initial dimeric aggregates based on CMD simulations. (**A**) Representative plot of inter-chain residue–residue contact pairs along simulation time (simulation 1). Shadow area denotes the “flat stage” along simulation times. Blue line indicates the simulation time of typical snapshot; (**B**–**H**) For seven aggregation simulations: **top** panels: the inter-chain residue contact frequency map based on shadowed area shown in (**A**) and Figure S6. The red circles in the top panels of Figure 4B–D and F–H represent the common contact pattern with the N-terminal of one protein (mainly residue 5–10) bonded with the N-terminal residues of the other; **Middle** panels: typical snapshot illustrations. Gradually changing colors from blue to red represent proteins from N to C terminal, and the sticks of the mainchain correspond to the circle regions on the top panels; **Bottom** panels: the intra-chain residue contact maps of each protein on the middle panels, with top-left (blue solid squares) and bottom-right triangles (red solid squares) representing chain A and chain B, respectively. To be clear, (**B**) is for simulation 1, and (**C**–**H**) correspond to simulations of (**A**–**F**) in Figures S3–S6, respectively.

To investigate how the aggregation occurred, we recorded the inter-chain residue contacts between the two monomers in dimeric aggregates. The contact was defined when any two non-hydrogen atoms in the residues from each monomer separated by less than 4.5 Å. Figure 4A indicates that the contact pairs varied with time evolution in simulation 1, and results of other simulations are shown in Figure S6. The plots indicated that, after the two proteins approached each other, the interfacial contacts between them varied, accompanying their conformation adjustments; some of the contacts remained, but others were broken (the white area within the points spreading region). With time evolution, the contact pairs in some simulations still remain unchanged, like the shadow areas in Figure 4A and Figure S6A,C,F; we called it the “flat stage.” In other simulations, they still increased (as shown in Figure S6B,D,E) but not as much as before, so we selected these shadow areas in the figures for further analysis.

The interfacial contact patterns formed in the dimeric aggregates were represented by inter-chain residue contact probability maps. The statistics were based on the conformations in the first 400 ns of shadow areas in Figure 4A and Figure S6. In six of the seven simulations, the N-terminal residues 5–10 of one of the monomers apparently bonded with the N-terminal residues of the other, as the circled areas show in the top panels of Figure 4B–H. In Figure 4C,H, the contacts from the other monomer were located at residues 5–10, while, in Figure 4D,F,G, they were located at residues 10–15. In Figure 4B, the two N-terminals packed into an antiparallel β-sheet with residues 1–10 from each protein. This common pattern provides important information for the DS119 unique folding behavior, as the segment of residues 5–10 is exactly the region of β-strand in the native structure. We further measured the number of hydrogen bonds formed between the packed segments in the circled areas, and the results indicated that at least one hydrogen bond was formed between the mainchain and lasted 600 ns to 1 µs. Thus, if the proteins in the aggregates further fold to its native structure, they have to break the hydrogen bonds, which would take more time to break free and then interact with its C-terminal to pack into the native parallel β-sheet.

### 2.4. DS119 in Initial Dimeric Aggregates Stabilized in the Intermediate States

For the seven aggregated simulations, we obtained the average structural frames of the aggregates corresponding to the shadow areas of Figure 4A and Figure S6. Furthermore, a typical photographed conformation with the smallest RMSDs from average structures (corresponding to the time labeled by vertical blue lines in Figure 4A and Figure S6) were extracted from the simulations, as the schematic illustrations demonstrate in the middle panels of Figure 4B–H. The sticks of the mainchain in the illustrations correspond to N-terminal contact patterns circled in the top panels of Figure 4B–H.

**Figure 5:**
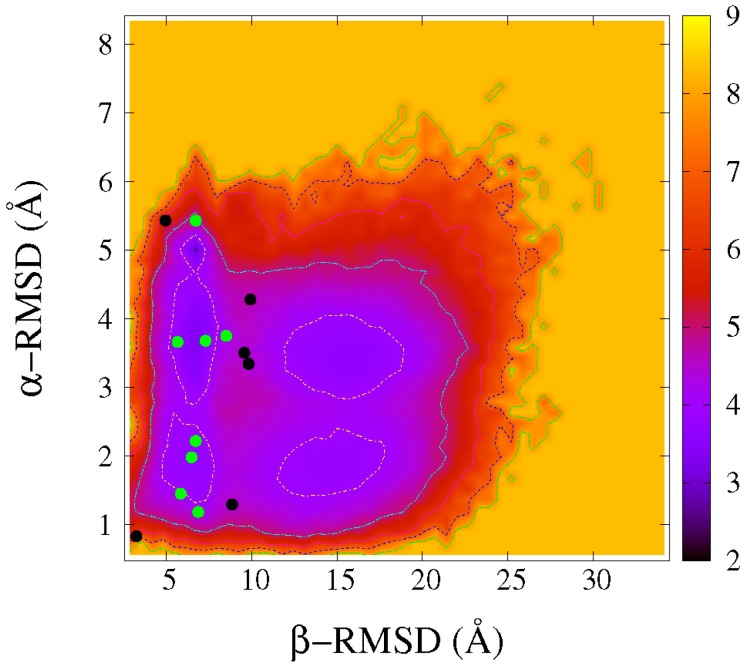
Locations of the typical conformation of monomers from dimeric aggregates on the free-energy landscape in Figure 2A. Green points represent those in or near the minimal regions and black ones represent those that are far from these regions.

Except for the exhibition of the features of the contact interface of the aggregates, these typical conformations also reveal monomer structural details. The intra-chain residue contact maps of each monomer are displayed in the bottom panels of Figure 4B–H. The top-left and bottom-right triangular areas denote each monomer in the corresponding aggregate. As we can see, in many of the monomers, the α-helix was formed better or worse, as indicated by the points near the diagonals. Meanwhile, the points in the top-left and bottom-right corners indicate the N- and C-terminal interactions. Compared to the native contact map shown in Figure 1B, it was apparent that the β structure was not formed. This is consistent with the secondary structure analysis mentioned above. To assess how similar these typical conformations are to those in the populated states in the REMD simulations, the β-RMSD and α-RMSD of each monomer in the typical structures were calculated. The locations of these conformations on the folding free-energy landscape at 319.3 K are shown as the points in Figure 5. Among them, one monomer, chain A in Figure 4G, was the most native-like and was located in the bottom-left corner, while its near-C-terminal segment formed a small nonnative α-helix, as shown in Figure 4G. As for the other points, most of them were located in or very near the F’, I1, and I2 regions, as indicated by the green points, four of which are in or nearby the F’ region, one of which is nearby the I1 region, and three of which are in or nearby the I2 region. This implies that these monomers in the dimeric aggregates would be stabilized in the intermediate states, and might take some time to disassociate from each other and then fold to their monomeric native structures.

## 3. Discussion

Most natural globular proteins with a single domain fold in a two-state manner, and it has usually been deemed the result of biological evolution that misfolding is avoided. However, *de novo* designed proteins are not subjected to natural selection, and much more attention is usually paid to their thermodynamic stabilities during the design strategy [16,35]. If they can fold similarly to natural proteins, this needs more investigation. DS119 was found to fold uniquely and slowly compared to natural proteins with a similar amino acid number, which might be due to transient dimers observed during the folding process [26]. In this work, we combined the REMD and CMD simulations to attempt to discover the mechanism behind this phenomenon.

Rational selection of order parameters are important for the analysis of free-energy landscapes based on REMD simulations, such as in the present work, *R*g-RMSD profiles displayed only two states, while β-RMSD and α-RMSD profiles, as well as those with W9–W34 distance and helix content, clearly showed several populated states. These rough landscapes would cause protein to halt in the metastable intermediate states during the folding process. For DS119, intermediate states observed here usually have a helix formed at different degrees, with the two terminal parts approached (at lower temperature) or not (at higher temperature). These conformational features of the intermediate states were also observed in the folding process based on long CMD simulations of the work of Qi *et al*. [25]. The free-energy landscape obtained by Hansmann *et al.* [36], using multicanonical molecular dynamics, also showed that the populated intermediate states formed with different helicities. Basically, the formation of the helix is easier and much more stable, as it is formed by local contacts. Metastable states with helices at lower degrees might arise from the small hydrophobic core, and the formation of the parallel β-sheet is difficult to stabilize it. Furthermore, in the metastable intermediates at low temperature, the N- and C-terminal nonnative interacts were observed (contact maps in Figure 2B,D,E), which should be the key to impeding the formation of parallel β-sheet.

The life span of the transient dimer in the folding of DS119 lasted an instant from milliseconds to seconds in the experiments; it is much beyond the computational ability of all-atom molecular dynamics simulations to detect the entire association-disassociation process. Therefore, the CMD simulations in this work could only detect the very initial association stage to discover how the transient dimer was formed. The observed common interfacial contact pattern and the monomer structural feature in the aggregates demonstrated that the N-terminal residues 5–10 of DS119 are not only prone to bond with the other monomer, but apt to form nonnative bonds with the C-terminal. This means that DS119 could be stabilized into dimeric aggregates. Therefore, this segment makes the protein difficult to fold via self-assembly.

**Figure 6:**
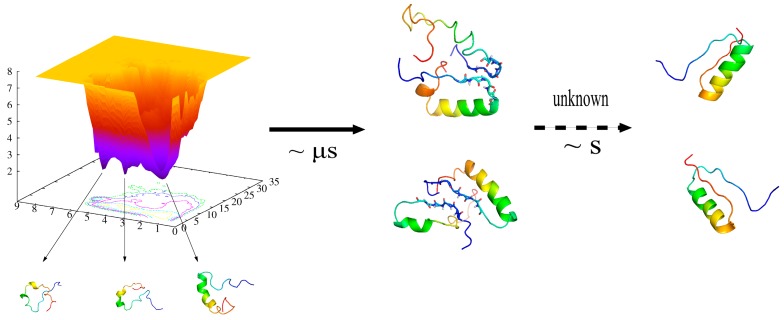
The schematic of possible folding pathway of DS119.

Based on the simulation results of this study and the reports of recent experiments [26], the possible folding pathway of DS119 was deduced as shown in Figure 6—unstructured DS119 is easily trapped in different intermediate states; then, under diffusion, two DS119 proteins form a transient dimer with a common binding interface at N-terminals, which occurs at a time scale of µs. The following dimeric aggregate dissociation and subsequent folding into native structures occurs in mere seconds based on experimental results, and a further investigation of how this occurs would provide us with a more complete understanding of the folding process. The complex folding of DS119, together with the studies of other *de novo* designed proteins, such as FSD-1 [17,18], α3D, and top7 [19,20,21,22,23], implies a current limitation of protein design, since they cannot fold like natural proteins to avoid intermediate states and aggregation.

In fact, homodimer is a very common type of protein assembly in nature, and there have been some successful designs of stable homodimers based on a monomer scaffold [15,37]. Moreover, in a report by Zhu *et al*. [26], a mutant of DS119 with three changed residues (V5E, T13D, and K21L), referred to as DS103, demonstrated folding into a stable dimer. By comparing DS119 and DS103, more investigations to discover how proteins fold from a transient dimer to a stable dimer, might be helpful for understanding protein structural evolution and provide new insight into rational protein design.

## 4. Materials and Methods

### 4.1. Replica Exchange Molecular Dynamics Simulations

The REMD simulations were performed using the AMBER14 package [38]. The AMBER 99SB force field [39], the continuum generalized Born, and the solvent accessible surface area (GB/SA) implicit solvation model were adopted [40,41]. To generate the initial conformations for all replicas, a fully extended structure was built for energy minimization with 500 steps via steepest descent and 500 steps via conjugate gradient algorithms, followed by a 500-ps molecular dynamics simulation at 500 K. Then, the obtained conformation was used as the starting structure for 26 replicas, with temperatures ranging from 260 to 517.2 K. The SHAKE algorithm was conducted to constrain the bonds involved with hydrogen atoms [42]. The simulation time step was 2 fs, and the data were recorded every 2 ps. Adjacent replicas were checked if they meet the exchange condition every 4 ps, and the average exchange rate was approximately 0.3–0.5 for all the replicas. The simulation time of each replica lasted for 500 ns; thus, the total simulation time for all 26 replicas was 13 µs. For each replica, the system potential approached equilibration after 50 ns; hence, the replica trajectories from 50 to 500 ns were used for analysis.

### 4.2. Conventional Molecular Dynamics Simulations

To investigate how the two DS119 proteins aggregate, eight independent conventional molecular dynamics simulations (CMD) were conducted by means of the Gromacs4.6.5 package, with an explicit solvent model TIP3P and AMBER99SB force field [43]. For each simulation, the initial structure comprised of two DS119 proteins separated at least 10 Å, and each of them was randomly selected from a 10-ns simulation trajectory performed at 550 K, starting from an extended conformation. Two proteins were solvated into a water box with a size of 10 × 10 × 10 nm^3^, and 4 chloride ions (Cl^−^) were added to maintain the electric neutrality. The total number of atoms in simulated box was around 32,800, with a concentration of approximately 0.03 mol/L. The SHAKE algorithm was used to constrain all covalent bonds. The particle-mesh Ewald algorithm (PME) was implied to account for long-range electrostatic interactions [44]. The cut-off value for the PME and the non-bonded van der Waals interaction was 10 Å. All the CMD simulations were performed at 300 K with the pressure at 1 bar in the NPT ensemble, and the Berendsen algorithm was used for the pressure coupling [45]. Each simulation was performed for 1 µs firstly, and, for those dimer aggregations observed, the simulation time was then extended to assure the aggregated time was no less than 1 µs. In total, the simulation time for all the CMD simulations was 10.7 µs.

### 4.3. Structural Details in the Simulations

The first model of the DS119 NMR structures (PDB ID: 2KI0) was selected as the reference for the root mean-square deviation (RMSD) calculation. The mainchain atoms of the α-helix (14–27 residues) and β-sheet (5–10 and 29–34 residues) (shown in Figure 1A,B) were selected for the RMSD calculations, as the other parts of the protein are quite flexible. In the present study, we also monitored the RMSDs of segments α-helix and β-sheet, labeled as α-RMSD and β-RMSD, respectively. Hydrophobic core used for calculating the solvent-accessible surface area in REMD simulations was defined including Ile8, Val10, Leu17, Leu20, Ala24, Ile29, Val31, and Phe33, as in [22]. In this report, all the schematic illustrations were obtained from Pymol [46].

## 5. Conclusions

In conclusion, we conducted all-atom REMD simulations on monomer and eight CMD simulations on dimers to investigate the possible reason for the unique slow folding of *de novo* designed DS119. The folding free-energy landscapes obtained by REMD indicated several populated states, including the I1, I2, and native-like state F’ in folding condition and the I3 and unfolded state U in unfolding condition. In the states of F’, I1 and I2, part helices were formed and the N- and C-terminal contacted nonnatively. The free-energy barriers between these states were marginally stable in thermodynamics, with values lower than 1.0 kcal/mol. This indicates that, in the folding process, DS119 might have a large chance of being kinetically trapped in intermediate states. The dimer aggregates emerged in seven of the eight CMD simulations, and most of them had a binding contact pattern in which N-terminal residues of one monomer, especially residues 5–10, were packed with N-terminal residues of the other monomer. This binding segment is supposed to form a parallel β sheet with the C-terminal segment of its own. Furthermore, the monomer structures in the dimeric aggregates were mostly located in the intermediate state regions on the folding free-energy landscapes. This means that the formed transient dimer would stabilize the monomer for a longer time, which might cause its unique, long, and complex folding behavior. Taken together, nonnative interactions play important roles for the formation of intermediate states and aggregation in the folding of *de novo* proteins, which implies that more evolution information should be included in rational protein design strategy to avoid this. By the mutation of the N-terminal segment (especially residues 5–10), or by using a small ligand to destroy the binding interface, it might be possible to prevent aggregation from occurring. This could provide a new system model to intervene with those diseases associated with protein aberrant folding.

## Supplementary Materials

Supplementary materials can be found at http://www.mdpi.com/1422-0067/17/5/612/s1.
