# Supplementary material for: The Folding of de Novo Designed Protein DS119 via Molecular Dynamics Simulations
Source: Int J Mol Sci. 2016 Apr 26;17(5):612. doi: 10.3390/ijms17050612 (PMC4881441; doi:10.3390/ijms17050612)
Supplement: Supplementary file 1 [file ijms-17-00612-s001.pdf]

Supplementary Materials: Folding of *de Novo* Designed Protein DS119 by Molecular dynamics Simulations

Moye Wang, Jie Hu and Zhuqing Zhang

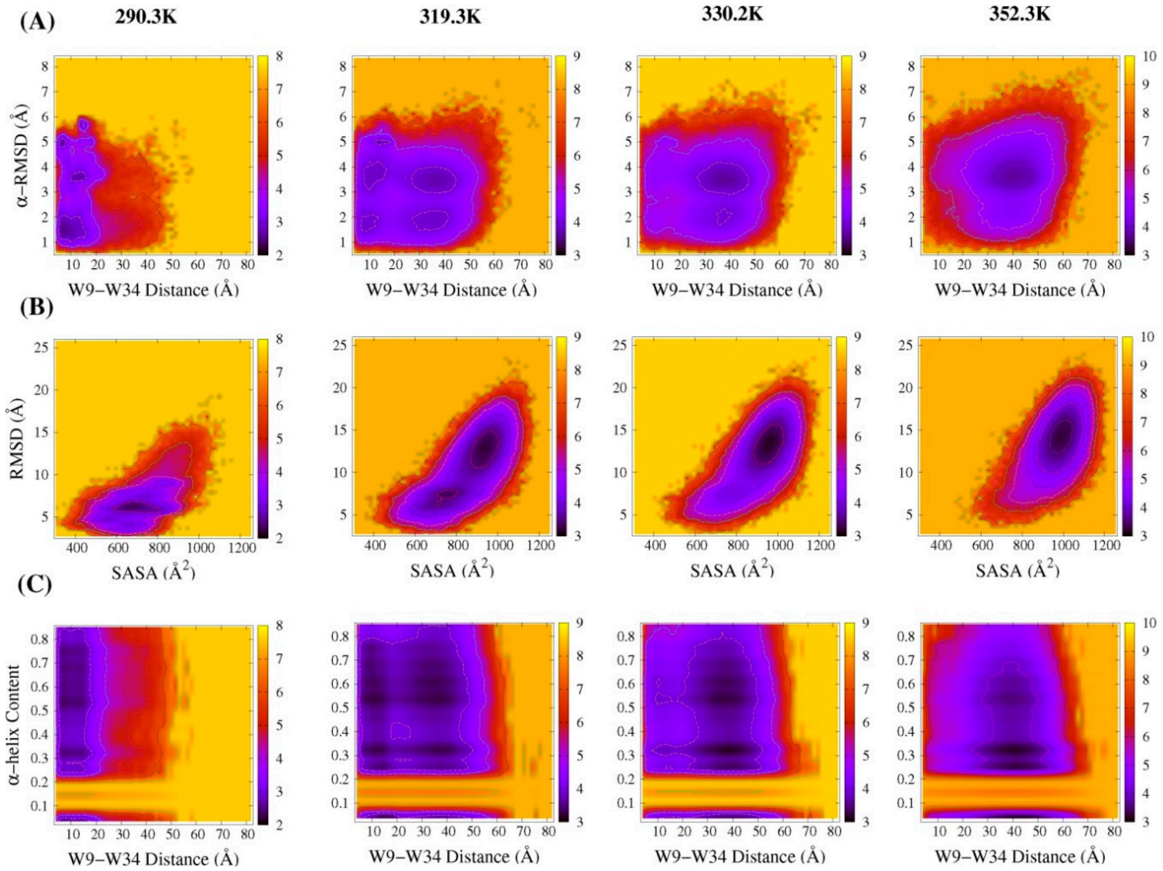

Figure S1. The free energy landscapes of DS119 with different reaction coordinates. (**A**) Reaction coordinates are α-helix root-mean-square deviation (RMSD) and the distance between aromatic groups of Trp9 and Trp34 (W9–W34 distance); (**B**) Reaction coordinates are the content of α-helix and W9–W34 distance; (**C**) Reaction coordinates are the RMSD of the whole protein and the solvent-accessible surface area (SASA) of the hydrophobic core. From left to right the temperature are 290.3, 319.3, 330.2 and 352.3 K respectively for (**A**–**C**).

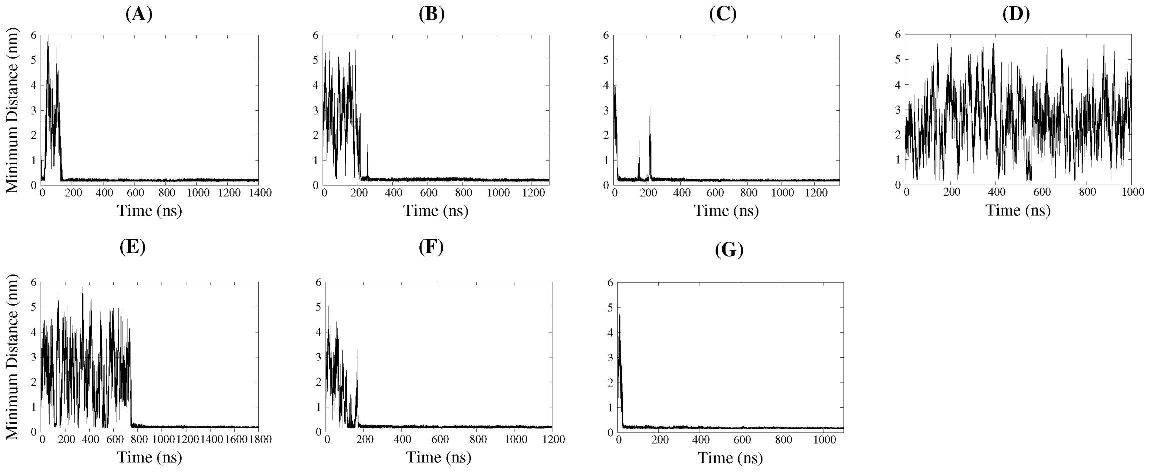

Figure S2. The minimal distance of two proteins along with simulation time in seven conventional molecular dynamics (CMD) simulations except simulation 1 which was shown in Figure 3B. (**A**–**G**) stands for the simulations 2–8 in the CMD in Figure 3B.

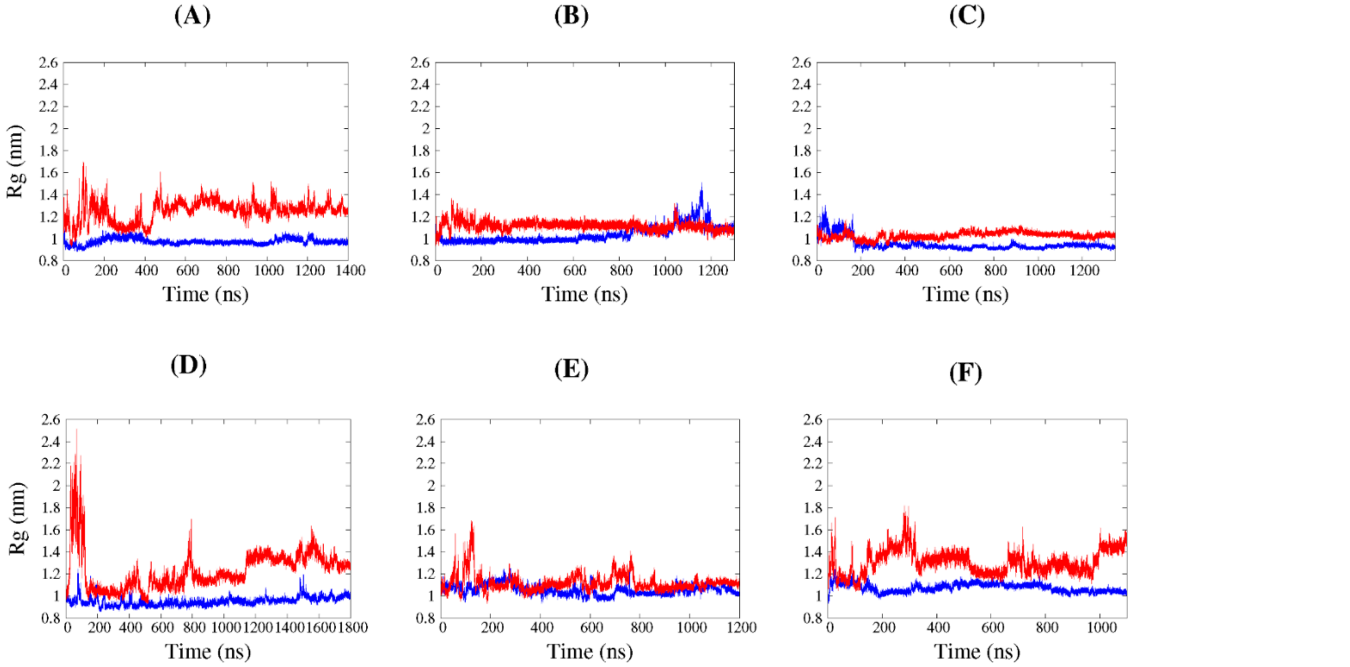

Figure S3. *Rg* values of two proteins with simulation time in six aggregated CMD simulations except simulation 1 which was shown in Figure 3C. (**A**–**F**) stands for the simulations 2–4 and 6–8 in which the aggregation occurred in the CMD in Figure 3C.The blue lines denote chain A and the red denote chain B.

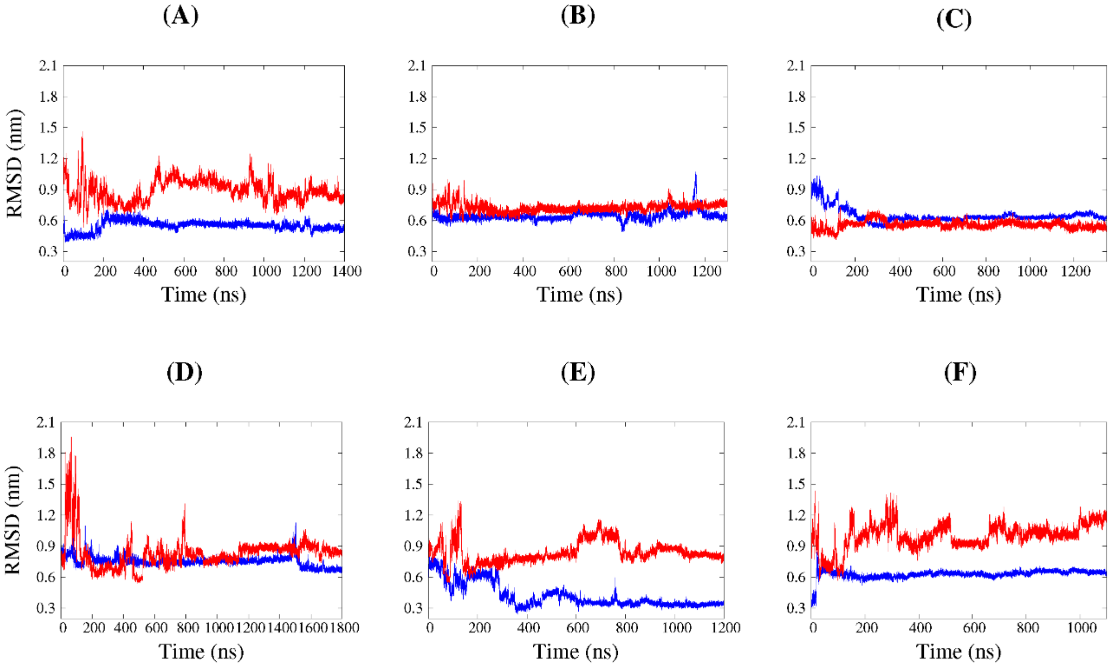

Figure S4. RMSD values of two proteins versus simulation time in six aggregated CMD simulations except simulation 1 which was shown in Figure 3D. (**A**–**F**) stands for the simulations 2–4 and 6–8 in which the aggregation occurred in the CMD in Figure 3D. The blue lines denote chain A and the red denote chain B.

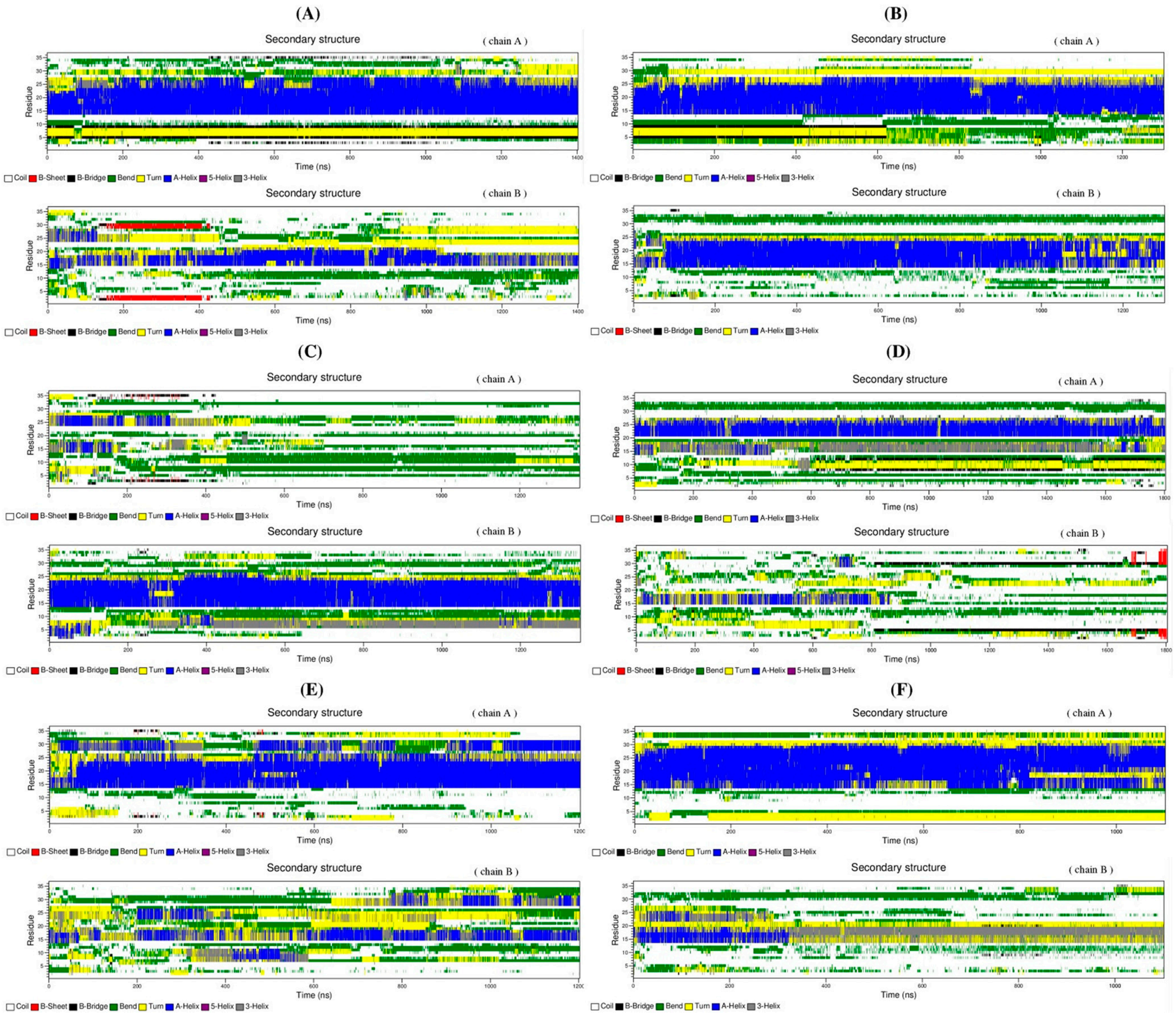

Figure S5. Secondary structure analysis (based on DSSP) of two proteins along with simulation time in six aggregated CMD simulations except simulation 1 which was shown in Figure 3E. (**A**–**F**) stands for the simulations 2–4 and 6–8 in which the aggregation occurred in the CMD in Figure 3F.

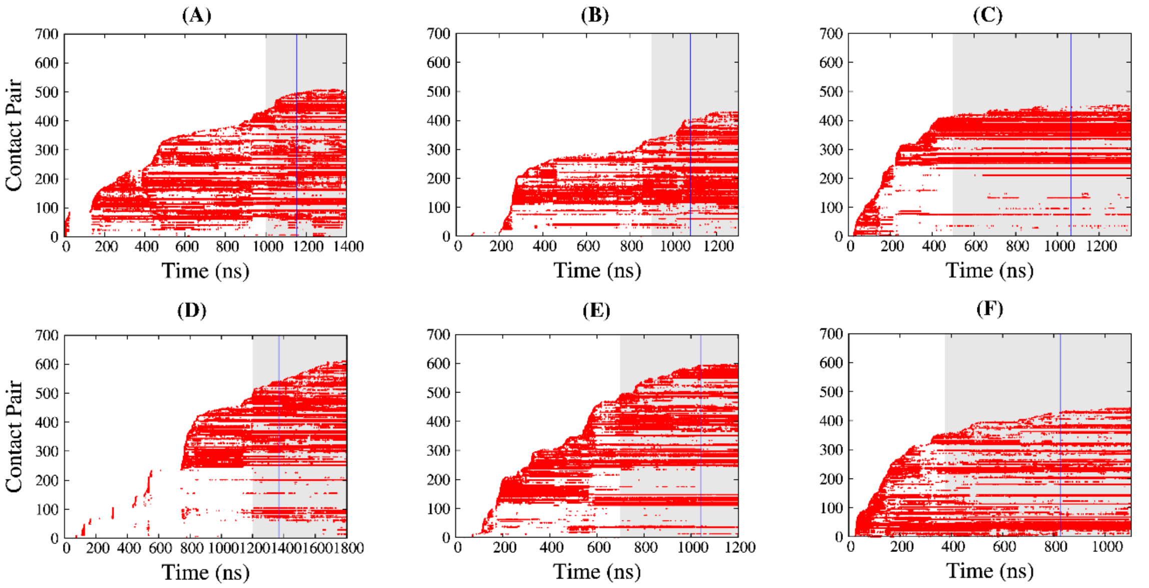

Figure S6. (**A**–**F**) Inter-chain residue contact pairs along simulation time for the six aggregated CMD simulations except simulation 1 which was shown in Figure 4A. Shadow areas denote the regions for statistical analysis. Blue lines indicate the snapshot times of representative structures (shown on the middle panels of Figure 4C–H).
